# Sequential information in a great ape utterance

**DOI:** 10.1038/srep38226

**Published:** 2016-12-02

**Authors:** Pawel Fedurek, Klaus Zuberbühler, Christoph D. Dahl

**Affiliations:** 1 Institute of Biology, University of Neuchâtel, Neuchâtel, Switzerland; 2 Budongo Conservation Field Station, Masindi, Uganda; 3 Max Planck Institute for Evolutionary Anthropology, Department of Primatology, Leipzig, Germany; 4 School of Psychology and Neuroscience, University of St Andrews, Scotland, UK

## Abstract

Birdsong is a prime example of acoustically sophisticated vocal behaviour, but its complexity has evolved mainly through sexual selection to attract mates and repel sexual rivals. In contrast, non-human primate calls often mediate complex social interactions, but are generally regarded as acoustically simple. Here, we examine arguably the most complex call in great ape vocal communication, the chimpanzee (*Pan troglodytes schweinfurthii*) ‘pant hoot’. This signal consists of four acoustically distinct phases: introduction, build-up, climax and let-down. We applied state-of-the-art Support Vector Machines (SVM) methodology to pant hoots produced by wild male chimpanzees of Budongo Forest, Uganda. We found that caller identity was apparent in all four phases, but most strongly in the low-amplitude introduction and high-amplitude climax phases. Age was mainly correlated with the low-amplitude introduction and build-up phases, dominance rank (i.e. social status) with the high-amplitude climax phase, and context (reflecting activity of the caller) with the low-amplitude let-down phase. We conclude that the complex acoustic structure of chimpanzee pant hoots is linked to a range of socially relevant information in the different phases of the call, reflecting the complex nature of chimpanzee social lives.

The acoustic structure of animal vocalisations is shaped in ways that effectively fulfil their functions^1^,^2^,^3^. In many species, calls reveal attributes intrinsic to the caller, such as identity, sex, age, body size, reproductive status, or social dominance^4^,^5^. Calls often facilitate the prediction of behaviour, which enables recipients to choose appropriate responses^3^,^6^,^7^. For example, signalling one’s own activities can be beneficial since it helps others to coordinate their own behaviour with those of other group members^8^,^9^,^10^. Animals can also inform others about extrinsic events, such as by producing distinct calls during feeding, which attract others, or acoustically distinct alarm calls to different predators, which enables others to take adaptive antipredator decisions^11^,^12^.

On a proximate level, the acoustic structure of calls is determined by the shape, size and flexibility of the vocal apparatus, consisting of vocal folds and supra-laryngeal vocal tract, as well as the muscle fibers and innervations that govern their movements^5^. For example, fundamental frequency (F0) is determined by the vibration of the vocal folds (the ‘source’ according to the source-filter theory^13^), which depends on their size and thickness, the result of an individual’s sex, age, reproductive status, and other physiological attributes or processes^5^,^14^. Formant properties are, on the other hand, determined by the shape and size of supra-laryngeal vocal tract (the ‘filter’), which correlates with body size and age^5^,^15^.

Many animal calls are structurally complex (*sensu*^16^) comprising several acoustically distinct elements, but the adaptive function of such complexity is still a topic of debate. One hypothesis is that acoustic complexity has evolved to facilitate individual recognition, especially in large societies^17^. For example, in sciurid rodents (Sciuridae) and other animals, variation in individually distinct calls correlates with both communicative and social complexity^18^.

Another hypothesis is that call complexity is a product of sexual selection and reflects the caller’s quality (*sensu*^19^,^20^), the classic example being birdsongs^21^,^22^,^23^. Another example is the frog species (*Physalaemus pustulosus*), in which males increase the complexity of their calls when competing with each other, which is attractive to females^24^. Similarly, multi-call sequences in male Gelada baboons (*Theropithecus gelada*) are more attractive to females than simple utterances^25^.

A third hypothesis is that, similar to human language, complexity enhances communicative potential of calls by callers combining basic acoustic elements to generate more complex structures^26^,^27^,^28^,^29^,^30^,^31^ or by combining stand-alone calls into sequences^32^. For instance, in chimpanzees (*Pan troglodytes*), victim screams and ‘waa’ barks are stand-alone calls but they can also be produced in sequence during agonistic interactions, in which case they are directed at different audiences and have different functions^33^. Recently, there have been considerable research efforts to investigate combinatorial phenomena in animal communication, largely because of their potential relevance to the origins of human language^34^.

However, there are also numerous examples of acoustically complex animal calls that are stereotypic and have few or no combinatorial properties. Here, the hypothesis is that complexity allows a caller to signal multiple types of information simultaneously (e.g. the multiple information hypothesis^16^,^33^,^35^). A typical example is one type of birdsong where several distinct notes are produced in a stereotypic order, with particular notes often fulfilling specific functions^36^. Empirical examples are the “snarr” elements in male water pipit song (*Anthus spinoletta*)^22^ and the “rattle” elements in male barn swallow song (*Hirundo rustica*)^21^, which reflect dominance and are produced mostly in intra-sexual competitive situations. Similarly, in rock hyrax (*Procavia capensis*), distinct song elements, such as “chucks” and “snorts”, are linked to different information, such as age and social status, and males adjust the presence or rate of these elements during territorial displays^35^,^37^.

Acoustically complex but stereotyped calls have also been described in non-reproductive contexts, which raises questions about their communicative function. One of the most charismatic examples in the animal kingdom is the pant hoot call of chimpanzees. Pant hoots are acoustically complex in that they are comprised of four distinct phases, introduction, build-up, climax, let-down, given in this sequential order^38^ (Fig. 1A; see Supplementary Audio S1 for an example of a recording). The introduction consists of low-amplitude, low-frequency ‘hoo’ elements, which grade into a low-amplitude build-up phase, comprising short low-frequency inhalations and exhalations. The build-up then grades into a high-amplitude, high-frequency climax phase, comprising between one to several successive ‘screams’. The climax, finally, grades into a low-amplitude, low-frequency let-down phase, which acoustically resembles the build-up phase.

Chimpanzee pant hoots have been the target of much field research, but the focus has usually been on the climax part of the call, which is individually distinctive and can be linked to a caller’s social status^39^,^40^, facilitating individual recognition over long distances in a society with a high degree of fission-fusion dynamics^41^,^42^,^43^,^44^,^45^. There have also been suggestions that the acoustic quality of the climax differs between calls produced in travelling and feeding contexts, but the corresponding data are inconclusive^46^,^47^.

The other three call elements of the pant hoot have received relatively little empirical attention. It is possible that these low-amplitude phases function, at least in part, to communicate to nearby party members. For example, the build-up phase appears to play some role in coordinating chorusing, insofar as callers adjust the duration of this phase to the vocal responses of party members^48^. Nevertheless, compared to the climax, the other three phases are still poorly understood in terms of their communicative value.

The aim of this study was to employ state-of-the-art acoustic analysis to examine chimpanzee pant hoots in their entire complexity for the main types of information often linked to animal signals: caller’s identity, social status, context (i.e. travelling and feeding; an indicator of the event experienced by the caller), and age. We considered age, not only because of the physical changes of the vocal tract during maturation^5^, but also because chimpanzees are long-lived primates with life-spans of 50 years or more, suggesting large age-related individual differences in terms of, for example, stamina or physical condition (e.g. ref. 49). Age, therefore, is likely to be an important attribute in wild chimpanzees, in addition to social status.

Acoustic analyses of primate vocalisations are traditionally carried out by manually extracting a limited number of arbitrarily chosen acoustic parameters from spectrograms, a time-consuming and tedious process that is based on arbitrarily selecting variables for subsequent statistical analyses^40^,^42^. In all likelihood, however, the neural machinery devoted to processing vocal sounds does not usually deal with single acoustic parameters. Most animal vocalisations are complex acoustic structures, but neural resources are limited so natural selection may favour representational feature embedding to minimise the required computational effort. A biologically plausible way of auditory processing and classification is to respond to a subspace that represents most of the signal’s inherent variance, as vision research has suggested for human visual processing^50^,^51^. We used an algorithm that automatically extracted audio features, the so-called Mel Frequency Cepstral Coefficients (MFCCs), and then applied a supervised learning algorithm (Support Vector Machine (SVM), as known in machine learning) for classification.

We compared the four phases of a pant hoot in terms of the four examined kinds of information they are potentially associated with. If the multiple-information hypothesis applied to chimpanzee pant hoots, we would expect that the four types of attributes of the caller correlate differently with the four phases of the call. In addition, since males sometimes omit the build-up or the let-down phase in a pant hoot, we examined whether the omission of these elements was associated with caller’s age, rank, and context of call production. It is important to note, however, that we did not aim to test directly hypotheses regarding the function of pant hooting, but rather to explore attributes, both intrinsic and extrinsic to the caller, associated with the four phases of the call, and therefore the potential of the call to signal multiple information sequentially.

## Results

We used a Support Vector Machine (SVM), a supervised learning algorithm, to classify audio samples. A classification routine consists of training and testing separate audio samples, i.e. one subset of all samples for training and a different subset for testing. Training samples, in addition, contain a class label (e.g. ID1, ID2, etc.). From the training samples, SVM creates a model that best separates samples of different class labels. Testing samples are then used to determine the performance of the model on novel samples. In the following we apply this principle to the four kinds of attributes: ‘identity’ (e.g. ID1, ID2, … IDn), ‘age’, ‘social status’ and ‘context’.

### Attributes associated with the four phases of a pant hoot

**Figure 1:**
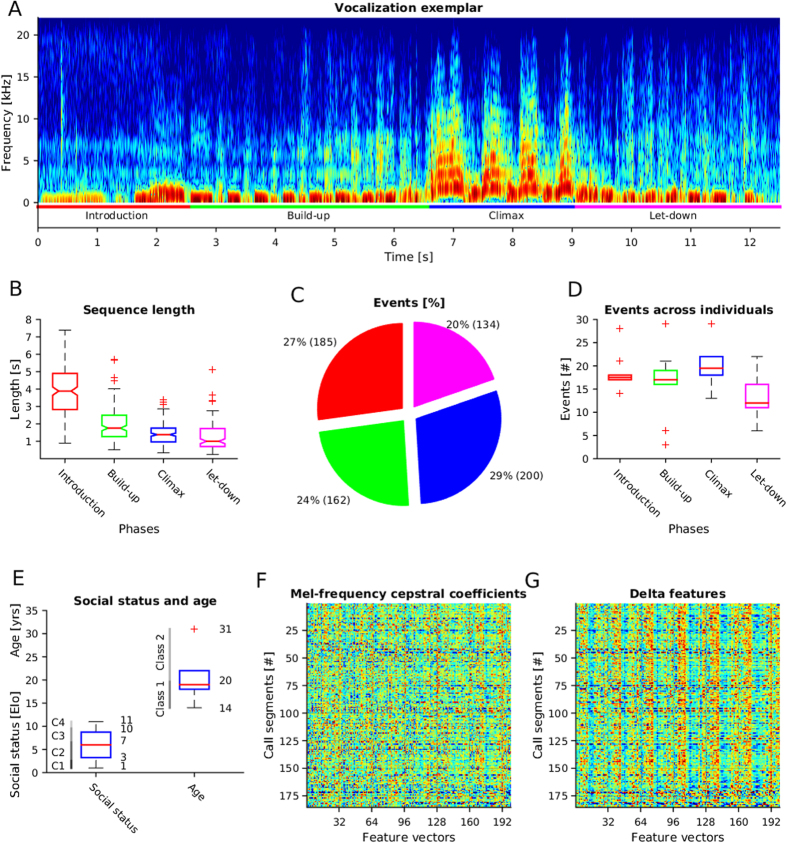
Four phases of pant-hoot and feature extraction. (**A**) An exemplar call sequence of a pant-hoot. The four phases of a pant-hoot exemplar are color-coded below the spectrogram: red: Introduction; green: Build-up; blue: Climax; magenta: Let-down. The x-axis shows the time in seconds; the y-axis shows the Frequency [kHz]. (**B**) Length of phases. The lengths of the four phases are shown on the y-axis of the boxplot. The color-coding is in correspondence with **A**. The boxes indicate the upper and lower quartiles and the median (red solid line). The whiskers indicate the highest and lowest values of 95% of the data samples. Outliers are indicated by red x-markers. (**C**) Number of recorded events. The number of recordings for each phase are presented in percentage and number count. Colors of pie chart patches correspond with the colors in (**A,B**). (**D**) Events across individuals. The number of recordings for each phase is shown across individuals. The y-axis shows the number count. Colors and markers as in (**B**). (**E**) Social status and age. Social status and age are shown across individuals. The y-axis has double scaling properties: Elo-ratings for social status and years for age. Classes as used for the classifier are indicated by labels like ‘C1’, ‘C2’, etc., or ‘Class 1’, etc. (**F**) Mel-frequency cepstral coefficients (MFCCs). MFCCs are shown as feature vectors (columns) for each call segment (rows). The x-axis has been arbitrarily limited to 200 samples for better illustration. (**G**) Delta features. Delta features are shown in the same way as the MFCCs in **C**.

In total, 681 phases from 221 calls were analysed (Fig. 1B–G).

#### Classification

**Figure 2:**
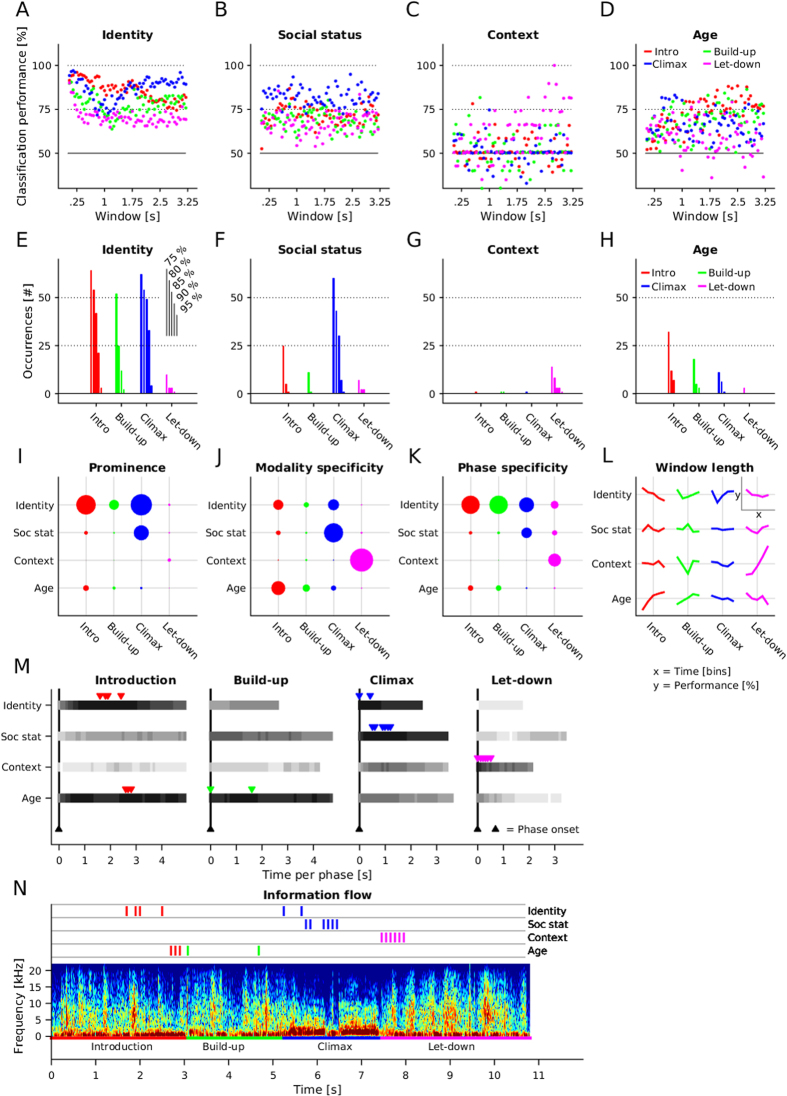
Classification performance. (**A**–**D**) The raw values of classification performance for each feature length and phase (dots in color-code; red: Introduction; green: Build-up; blue: Climax; magenta: Let-down). The x-axis is the feature/window length; the y-axis is the classification performance as percentage correct classification. (**E**–**H**) The number of occurrences in the range of 75 to 95 percentage correct classification. The x-axis shows the number of occurrences. (**I**) Prominence. The effect sizes are illustrated as filled circles with radii corresponding to the effect sizes. (**J**) Modality specificity. The relative contribution of each modality (caller’s attributions) in a particular phase is shown as filled circles with respective radii. (**K**) Phase specificity. The relative contribution of each phase to a particular modality (caller’s attributions) is shown as filled circles with respective radii. (**L**) Window length. Classification performances as in (**A**–**D**) were binned into five classes to illustrate the relative importance of window lengths for classification. The x-axis shows the phases at the global level and the time of the window bins on the local level. The y-axis shows the modality at the global level and the average performance scores at the local level. (**M**) Time dependency. The five points in the time course of a call sequence are highlighted with downward pointing arrowheads. The x-axis illustrates the time in seconds aligned to the onset of each individual phase. The y-axis shows the modalities. (**N**) Information flow. We illustrate the progression of information over time. The x-axis shows the time in seconds of a whole pant hoot. The y-axis is restricted to the spectrogram example and represents the frequency (kHz).

We determined the percentage of correct classification for ‘identity’, ‘social status’, ‘context’, and ‘age’ in each phase (introduction, build-up, climax, let-down) and for a range of window feature lengths (see Methods, Fig. 2A–H).

We found that ‘identity’ was correlated with all four phases, but most strongly with the introduction phase, followed by the climax phase (red and blue dots, Fig. 2A). While shorter time windows accounted best for ‘identity’ in the ‘introduction’ phase, longer time windows accounted best for ‘identity’ in the climax phase. When looking at occurrences of correct classifications in the upper performance range (>75%), identity was again strongly correlated with the introduction and climax, less so with the build-up and least with the let-down phase (Fig. 2E).

‘Social status’ was associated most strongly with the climax phase (blue dots, Fig. 2B), irrespective of the window length, with the strongest relative contribution in the climax phase (Fig. 2F).

‘Context’ was associated almost exclusively with the let-down phase, but mostly with longer time windows of more than 1.5 s (Fig. 2C,G).

‘Age’ was also present throughout the signal, but most strongly so in the build-up and introduction phases (green and red dots, Fig. 2D) with the strongest relative contribution in the introduction, followed by the build-up and climax phases (Fig. 2H).

#### Prominence

For prominence, i.e. the number of times correct classification was higher than 75%, we found the largest effects for identity (introduction, climax), followed by social status (climax), while age (introduction, build-up, let-down) and context (let-down) were relatively less prominent (Fig. 2I).

#### Modality specificity

For modality specificity, i.e. the number of occurrences of percentage correct classification above 75% normalized to each modality (or the relative contribution of each phase to a particular attribute of the caller, with circle sizes in each horizontal line adding up to 100%), we found again that ‘identity’ was not very modality specific, but spread across all phases (Fig. 2J). For ‘social status’, modality specificity was highest in the climax phase, given the overall dominance of number of occurrences at all window lengths in the climax phase. For ‘context’, finally, modality specificity was highest in the let-down phase, with no other phase contributing to the effect. For age, modality specificity was strongest in the introduction, followed by build-up and climax. Overall, the greatest extent of modality specificity could be found in the climax phase (‘social status’) and the let-down phase (‘context’).

#### Phase specificity

Phase specificity reflects the relative contribution of each attribute (‘identity’, ‘age’, ‘social status’, and ‘context’) to the different phases (Fig. 2K). The dependent variable was the number of occurrences of percentage correct classification above 75%, normalized to each phase. Circle sizes in each column add up to the maximum (100% of occurrences). With this measurement, the relative contribution of caller’s attribute in the introduction phase consisted mainly of ‘identity’ and to a lesser degree of ‘age’ and ‘social status’. A similar distribution of information could be seen in the build-up phase. Also in the climax phase, the most prominent attribute was ‘identity’, aside from ‘social status’. In the let-down phase, the strongest attribute was ‘context’, followed by ‘identity’. Together, a great amount of phase specificity could be seen throughout the introduction, build-up and climax phases with the dominance of ‘identity’, as also seen in Fig. 2I (Prominence). Phase specificity could also be found in the let down phase with ‘context’ apparent in longer window sequences.

#### Window length

Window length represents the influence of the time period over which the feature extraction was carried out on the performance of the classifier (Fig. 2L). For each phase, the x-axis illustrates five bins of window lengths, while the y-axis represents the performance scores. ‘Identity’ seems to be associated mainly with short segments, ranging from 50 to 1000 ms in the introduction, while ‘age’ and ‘context’ are associated mainly with long segments, ranging from 1750 to 3250 ms (introduction, let-down, respectively).

#### Information flow

To determine the time point in the call sequence that was most associated with a given attribute of the caller, we re-ran the SVM for all phases and attributes and restricted the selection of features to a certain period on the time course of the call sequences (Fig. 2M,N). Among the time points contributing relatively the most to each attribute throughout the call sequence (all phases), we selected the five highest scores and marked their time points visually (Fig. 2M). The general trend as described above is reflected in terms of which phase is associated with which attribute. Of all four phases, the introduction tends to be the longest (Fig. 1B), with both ‘identity’ and ‘age’ associated most strongly with the middle of the phase. For the shorter build-up phase, ‘age’ was apparent most strongly early in the phase, while for the climax phase, ‘identity’ and ‘social status’ were apparent early on. For the short let-down phase, ‘context’ was apparent in very early stages (Fig. 2M).

##### Composition of pant hoot calls

Not all pant hoots produced by chimpanzees consisted of all four phases, and especially the build-up and let-down phases were occasionally omitted. We found that the presence or absence of build-up or let-down was not associated with a caller’s social rank (GLMMs: β ± SE = −0.12 ± 0.21, z = −0.61, *P* = 0.542 and β ± SE = −0.06 ± 0.57, z = −1.02, *P* = 0.309, respectively) or age (β ± SE = 0.17 ± 0.14, z = 1.21, *P* = 0.228 and β ± SE = −0.03 ± 0.03, z = −1.91, *P* = 0.360), but males were significantly less likely to produce complete pant hoots in feeding than travelling contexts (build-up: 95.58% and 73.17%; let-down: 93.52% and 54.88%, travelling (N = 82) and feeding (N = 139) contexts respectively; build up: β ± SE = −1.82 ± 0.64, z = −2.84, *P* = 0.004; let down: β ± SE = −2.47 ± 0.41, z = −5.99, *P* < 0.001).

## Discussion

Male chimpanzee pant hoots usually consist of four phases: the introduction, build-up, climax and let-down, produced in succession. While previous studies focused predominantly on the function and information associated with the climax, here we were able to show that all four phases of the pant hoot may be associated with a variety of information that differ considerably between particular phases. Our results are consistent with the view that the complex acoustic structure of a pant hoot, with its four distinct phases, is shaped in a way that enables the receiver to acquire several kinds of information from different parts of the call.

In particular, the identity of the caller was most strongly correlated with both the low-amplitude introductory and the high-amplitude climax phases, reflected in the amount of prominence and modality specificity. Importantly, identity information seems to be not very phase-specific, showing strong association with the introduction, build-up, climax, but not let-down, phases.

This result is consistent with the view that the call functions to influence the behaviour of receivers not only in other parties, but also of those in close proximity^52^. More specifically, while the individually distinctive climax part of the call may be informative with regard to the caller’s identity for individuals in other parties (e.g. ref. 42), the introduction and build-up phases likely inform about this particular attribute individuals in the caller’s or a nearby party. Pant hoot chorusing plays an important role in signalling social bonds between males^52^,^53^ and males usually join in another male’s pant hoot at the introduction or build-up stage^48^. Therefore, signalling the identity of the caller at the early stage of the call may facilitate chorusing by allowing allied males to join the call before it progresses into the climax. This may be relevant to chimpanzees, where males, although in the same party, are often not in visual contact because of dense forest vegetation or due to being involved in activities such as grooming or feeding (P. Fedurek, pers. observation).

Our results show that it is not the high-frequency climax but the low-amplitude let-down phase that correlated most with caller’s activity. We found an overall relatively weak prominence for a caller’s activity, indicating that activity information is a very subtle modification of acoustic parameters in the call, but relatively strong modality and phase specificities. This suggests that ‘activity’ information is associated *only* with the let-down phase. Whether these context correlates of the let-down phase (which, on a proximate level, may be by-products of the activity of the caller, e.g. moving versus resting^47^) influence the behaviour of receivers, remains to be examined.

We showed that pant hoots produced in feeding contexts were less likely to contain both the let-down^47^,^54^ and build-up phase than calls produced in travelling contexts. These structural differences may well be informative for receivers regarding the general activity of the caller, arguably more so than the spectral properties of the call. The information on the feeding activity of the caller may therefore be associated with the call in at least two ways: by the acoustic structure of the let-down as well as by including or omitting this phase. The let-down is not always omitted in the feeding context possibly because this phase also serves other functions than signalling the activity of the caller. Since age was correlated mainly with the build-up phase, pant hoots without this phase are probably less likely to be associated with such information.

Our results are consistent with earlier analyses showing that social status is one of the functions of pant hooting^45^,^55^, and that a caller’s social standing is mainly reflected in the call’s loud climax^39^,^40^. We found relatively strong modality and phase specificities for ‘social status’, which supports this notion. Indeed, as mentioned before, some of the acoustic properties of a pant hoot climax, such as its initial peak frequency, correlate with testosterone^40^, a hormone that in many animals mediates the production of calls involved in intra-sex competition^56^,^57^,^58^, and, in chimpanzees, reflects social status and potentially male quality^59^,^60^.

Age was most strongly correlated with the introduction and particularly with the build-up phase. Although signalling age may not be particularly adaptive during intra-community interactions where all individuals know each other, it is possible that this is more important during close range inter-community interactions. For example, pant hoots of males in their prime may be aversive to neighbouring communities. Interestingly, males often prolong the build-up stage and omit the climax part of pant hoots when exchanging calls with adjacent communities (e.g. ref. 61; P. Fedurek, pers. observation), as if signalling their age but not social status. However, it is unclear whether age correlates of pant hooting, which most likely result from physical properties of the vocal tract that change during maturation (e.g. ref. 5), constitute a signal.

Our study lends support for the hypothesis that one of the reasons for a complex structure of chimpanzee pant hoots is that it enables receivers to gain several kinds of socially important information from a single call. Whilst it has been suggested that combining different call types^62^ or graded vocal systems^33^ enhance the communicative capacity of species with limited vocal repertoires, we propose that partitioning calls into distinct segments that are independently associated with different attributes of the caller, may be another way of overcoming this limitation. It has been shown before that in some bird species, especially those with limited vocal repertoires, different elements within the same stereotyped song have different functions^36^,^63^. For example, ‘complex note’ elements in white-crowned sparrow (*Zonotrichia leucophrys pugetensis*) songs seem to signal caller’s identity, while the trill element signifies motivation or reproductive state, both of which facilitate territorial defence^36^. Similarly, specific elements in the rock hyrax songs signal independently information on body size, weight and condition, and social status^35^. In some avian and mammal species, callers modify the presence or rate of call elements responsible for driving away sexual rivals^21^,^22^,^35^. We have shown, however, that the multiple information hypothesis may apply not only to calls linked predominantly to territorial or reproductive contexts. Particular elements within the same and highly stereotyped chimpanzee call, for example, can signal independently a variety of socially relevant information, which is consistent with the multiple social functions associated with pant hooting ranging from regulating grouping patterns to mediating social bonds^45^,^64^.

Capitalizing on the knowledge from previous research on chimpanzee pant hooting, the aim of this study was to examine the kind of information particular pant hoot phases are potentially associated with, by exploring their biologically relevant correlates. Our approach is therefore an indirect way of looking at the function of these phases. Examining whether or not these correlates are relevant for the receiver would require examining its responses to particular call parts in controlled experimental conditions. However, conducting playback experiments on wild chimpanzee populations using long-distance calls is extremely challenging^65^. Testing the multi-information hypothesis would also require playing back to a subject chimpanzee a single phase of the call rather than the whole pant hoot, which, at least for some phases, would lack ecological validity. Nevertheless, as mentioned above, our results are consistent with the substantial body of literature exposing the functional versatility of this call.

Our findings might be relevant to other apes producing calls with discrete, although less distinct than in chimpanzees, phases, such as orang-utan (*Pongo spp.*)^66^ and some gibbon (*Hylobates spp.*) calls^67^. As in the case of chimpanzee pant hoots, white handed (*Hylobates lar*) or agile gibbon (*Hylobates agilis agilis*) songs contain introductory and climax phases in their acoustically stereotyped loud calls. Furthermore, similarly to chimpanzee pant hooting, both the introductory and the climax phases of their long calls are individually distinctive^68^. While the function of the climax is to locate neighbouring conspecifics^69^ or to repel sexual rivals^70^, the introduction seems to be directed at nearby individuals, usually family members, to facilitate chorusing (e.g. ref. 71).

In conclusion, our study is consistent with the multiple information hypothesis. By virtue of being a complex, multi-phased call, different phases of the call seem to co-vary differently with specific attributes of the caller (such as identity, age, or social status) or with context of call production. This consistent covariance might then allow receivers to make decisions about subsequent responses. In contrast to avian singing, however, the multi-information quality of chimpanzee pant hooting seems to have functions beyond territorial or reproductive displays, which likely reflects the complex nature of a chimpanzee society. A noteworthy point is that the novel methods employed in this study allows for arguably more objective and more accurate way of analysing calls in terms of intrinsic and extrinsic attributes associated with them than the traditional methods often based on manual acoustic feature extraction.

## Methods

### Study site and study subjects

The study was carried out with the Sonso chimpanzee community (*P. t. schweinfurthii*) of Budongo Forest, Uganda. The group has been studied since 1990 and is well habituated to the presence of human observers^72^. At the time of the study, the community contained 75 individuals, including 14 adult and late adolescent males, and occupied a home range of around 15 km^2^. Subjects were adult (*N* = 8: ≥16 years) and late adolescent (*N* = 2: 13–15 years^73^) males (see Supplementary Information and Table S1 for age and rank information).

### Sampling method

Data collection methods for this study were strictly non-invasive and were approved by, and carried out in accordance with, the Institute of Biology Ethics Committee at the University of Neuchâtel. The study was conducted in accordance with laws, rules and regulations governing animal research in the European Union. The study was approved by the Uganda Wildlife Authority and the Uganda National Council for Science and Technology.

The study was carried out between June and October 2013, February and September 2014, and January and October 2015. Data were collected between 7.00 am and 4.30 pm local time. A randomly chosen male was followed for a whole day. Vocalizations were audio-recorded from both the focal male and, if possible, all other males present in his party, using a Marantz Professional PMD661 solid-state recorder and a Sennheiser ME67 directional microphone. In addition, the context of pant hoot production (travelling or feeding) was noted.

### Data collected and definitions

#### Context

Pant hoots are predominantly produced in travelling and feeding contexts^45^. Pant hoots produced when arriving at a feeing site (e.g. approaching or climbing a feeding tree), or during feeding, were classified as pant hoots given in feeding context. Pant hoots produced by males when moving on the ground (as opposed to resting, arriving at a feeding site or feeding) were classified as calls given in travel context.

#### Social status

Social status was established for adult and late adolescent males, using the Elo-rating procedure. This method is based on a sequence in which interactions between individuals occur rather than on an interaction matrix (ref. 74; see Supplementary Information).

### Data preparation and analyses

Data analysis was performed using Matlab (Mathworks Inc., Natick, MA, USA).

#### Attributes associated with the four pant hoot phases

A call was defined as “pant hoot” only if it contained the climax phase^45^,^53^. For acoustic analyses, we cut all call recordings into parts representing the four phases of the call. The duration of a phase was defined as the part between the start of the first exhalation and the end of the last exhalation of that phase^48^. We then examined all recordings and incorporated in the analyses only those that were complete and of high quality without background noise. Recordings from the four phases were analysed separately.

#### Feature selection

Feature selection, in machine learning, is referred to as the selection of a subset of relevant features (variables or attributes) to be used in the construction of a classifier. Feature selection is inevitable when classifying audio signals due to the high amount of redundant and irrelevant information in unprocessed signals. Further, feature selection reduces computational efforts and enhances generalization by reducing overfitting. We here extracted features that can be reliably estimated from the available audio signals and are relevant for classification. We extracted multiple features under the assumption that classes cannot be described by a singular point but by the distribution of samples at various feature dimensions. We extracted the Mel-frequency cepstral coefficients (MFCCs). MFCCs have been state-of-the-art in speech recognition for many years^75^, but have only recently been applied to the analysis of animal calls (e.g. ref. 76). MFCCs represent the envelope of the short-time power spectrum of a sound determined by the shape of the vocal tract. To address the change of the signals across time, the signal is cut into subdivisions under the assumption that the changes are smaller within than between subdivisions. We subdivided the signals into 30 ms frames with 50% overlap and applied a Hamming Window to obtain reliable spectral estimates. We then computed the fast Fourier Transform (FFT) for each frame and applied a mel filterbank to the spectrum to estimate the entire amount of acoustic energy in the various frequency ranges. We extracted 32 spectral bands ranging from 1 to 20,050 Hz. The Mel filterbanks are as follows: [0.0016, 0.1248, 0.2479, 0.3711, 0.4943, 0.6174, 0.7406, 0.8638, 0.9869, 1.1101, 1.2332, 1.3564, 1.4796, 1.6027, 1.7259, 1.8491, 1.9722, 2.0954, 2.2186, 2.3417, 2.4649, 2.5880, 2.7112, 2.8344, 2.9575, 3.0807, 3.2039, 3.3270, 3.4502, 3.5734, 3.6965, 3.8197] *10^−3^. This converted back to Hertz results in the following values: [0.0001, 0.0082, 0.0172, 0.0273, 0.0385, 0.0511, 0.0650, 0.0806, 0.0980, 0.1174, 0.1391, 0.1632, 0.1902, 0.2202, 0.2537, 0.2911, 0.3328, 0.3793, 0.4312, 0.4891, 0.5537, 0.6257, 0.7060, 0.7956, 0.8956, 1.0071, 1.1315, 1.2702, 1.4250, 1.5976, 1.7902, 2.0050]*10^−4^. We then calculated the logarithm of the filterbank energies to more closely simulate primate hearing and applied discrete cosine transform (DCT) to convert the mel spectrum into cepstral coefficients^77^. To capture the dynamics between the cepstral coefficients we computed their first-order derivative, resulting in delta-cepstral coefficients.

#### Supervised learning algorithm

We implemented a support vector machine (SVM) (LIBSVM toolkit^78^) for the audio signal classification. A classification task includes a training and a testing phase with separate training and testing samples. A class label (target value) is assigned to each training sample. Each sample per se is a vector of attributes, or in other words, a number of features or variables. From the feature vectors of the training samples, SVM creates a model that best separates samples of different class labels. To solve this task, SVM projects the feature vectors of each training sample into a higher dimensional feature space and determines a plane (a so-called hyperplane) with maximal margin, i.e. the largest separation, between samples of different class labels. A standard procedure using SVM is to try to separate all samples of the two classes. The problem with this procedure is that “unusual” samples or samples with wrong labelling may lead to a poor model. To avoid this, a technique called “soft-margin” is used to allow some mistakes in the training phase, leading to a better overall fit of the model and to better generalization. The parameter C describes the soft-margin cost function, a function that evaluates error penalty in order to gain stability. Larger values of C are indicative for a smaller margin, and vice-versa. Further, while standard procedures use a linear type of classification via dot product, we here applied a non-linear kernel function using radial basis function with a free parameter Gamma. Small Gamma values are indicative for a Gaussian with a large variance, implying that the class of this support vector influences the decision on other samples even if the distance between them is large. With a large gamma, the variance is small, reducing the influence of the support vector to proximal space. After determining the model based on the training samples, the testing samples are used to evaluate its classification performances by mapping them into that same space and predicting the class label according to which side of the gap (margin) they fall on. We reported these values as percentage correct classification.

In our study, we used input vectors representing the multivariate information of audio signals (as described above) of n-milliseconds of length (while n = [50:50:3250]). In other words, while SVM requires feature vectors ought to be of similar lengths, we systematically varied the length of the input vectors. We then used feature scaling (range 0 to 1) and mean normalization. Both are processes that standardize the range of the feature values in order to optimize the classification performance. This step of preprocessing the data avoids a situation where, for example, one feature has a broader range of values than all other features and would determine distances in the feature space most strongly. The goal of feature normalization is to have an approximately proportional contribution of all features to the final distance. The SVM used a radial basis function Kernel (RBF). We further five-fold cross-validated the soft-margin parameter C and the Kernel parameter Gamma on separated smaller datasets (~10% of complete dataset). This procedure ensures an optimal compromise between high performance scores and low generalization errors, as described above. The range of the parameters for cross-validation were [2^−5^, 2^−3^, … 2^15^] for C and [2^−15^, 2^−13^, … 2^3^] for Gamma. The optimized parameters were determined prior to each of the main classification procedures to prevent data biases. We then trained the SVM with 80% of the remaining dataset and tested it on a hold-out set of 20%. Training and testing labels were of four types: identity (ID number of the caller; N = 10); age (age class 1: [14 to 19 years], N = 5 individuals; age class 2: [20 to 31 years], N = 5 individuals); social status (rank class 1: [1:3], N = 3 individuals; rank class 2: [4:6], N = 3 individuals; rank class 3: [7:9], N = 2 individuals; class 4: [10:12], N = 2 individuals); context (context 1: feeding (N = 82); context 2: travel (N = 139). We then tested the prediction of the SVM by training and testing on one particular class versus another class. For ‘identity’, calls produced by individual 1 (ID1) fell into the first class and calls produced by other individuals fell into the second class. Importantly, training and testing always followed the same set of labels, e.g. ‘identity’. The reported values are the results of averaging the percentage correct classification of all possible combinations of comparisons (e.g. ID1 versus others, ID2 versus others, etc. for each label (‘identity’, ‘age’, ‘social status’, ‘context’), time window ([50:50:3250] ms) and phase (introduction, build-up, climax, let-down) separately.

#### Percent correct classification

Prior to feature selection we extracted a call segment with predetermined length from the original call sequences. The length was systematically varied from 50 to 3250 ms with a 50 ms increment. Such segments were extracted at randomly selected points in time (relative to call onset) and in a non-overlapping fashion throughout the entire call sequences. Importantly, for the SVM procedure, only one segment of each original sequence was selected to avoid unwanted classification biases. In cases where the extracted segment length was longer than the original call the remaining positions were filled with ‘NaN’s.

We determined the percentage correct classification of the SVM for each caller’s attribute (i.e. ‘identity’, ‘age’, ‘social status’, and ‘context’) and each phase. Since the SVM compares two classes at the time, the baseline is at 50% correct classification. The overall scores were determined by averaging condition-wise (as described above) (Fig. 2A–D). Further, to illustrate the effect sizes of each attribute of the caller and each label, we determined how many times (number of occurrences) the SVM testing procedure yielded more than or equal as n% correct classification (while n = [75, 80, 85, 90, 95]). This is illustrated as bar graphs in Fig. 2E–H.

#### Descriptive indices

‘Prominence’ (Fig. 2I) illustrates the effect size of each caller’s attribute in each label considering all extracted segment lengths. ‘Prominence’ was determined by averaging the number of occurring events higher than 75, 80, 85, 90 and 95% correct classification. ‘Prominence’ is represented in the size of linearly scaled filled circles, with larger circles being more prominent than smaller circles. In a similar fashion we determined ‘Modality specificity’ (Fig. 2J) and ‘Phase specificity’ (Fig. 2K). While Modality specificity shows the relative contribution of each modality (caller’s attributes) in a particular phase, Phase specificity shows the relative contribution of each phase to a particular modality (caller’s attribute). Circle size represents the degree of specificity. For the Modality specificity, circle sizes in each row (e.g. Fig. 2J, all phases for Identity) add up to 100%; for the Phase specificity, circles sizes in each column (e.g. all caller’s attributes in the introduction phase) add up to 100%. Further, we average classification performances for each attribute of the caller and phase according to the segment length extracted for classification. We therefore split the segment lengths into five classes (class 1 = [50:700]; class 2 = [750:1350]; class 3 = [1400: 1950]; class 4 = [2000:2600]; class 5 = [2650: 3250], units: ms) and show the average values for each of these bins (Fig. 2L). We normalized the resulting values for each row (caller’s attribute). This illustration aims at pointing out the relative influence of segment length per each attribute and phase.

#### Time-dependent analysis

We re-ran the SVM analysis by extracting call segments at a given point in time (i.e. relative to the onset of the call). The segments were selected at the following points in time (ms): [0:100:3000]. Similarly as in the previous analysis, we systematically varied the length of the extracted call segment. We then averaged the data across all time segment lengths and displayed the five time points with the greatest average performance (see arrow heads in Fig. 2M).

### Presence and absence of phases within pant hoots

Chimpanzees sometimes produce incomplete pant hoots with one or more units missing, particularly the build-up and let-down phases. To examine whether there was a relationship between incomplete pant hoots and context, we created two General Linear Mixed-Effect Models in which we put as the dependent variable whether or not the call contained build up or let down phases, and with context (0: travel; 1: feeding), age and social rank as the independent variables. Analyses were carried out on N = 221 calls (travel: N = 139; feeding: N = 82). We used Praat (version 5.2.19) for acoustic analysis and Stata 12.0 (StataCorp LP, College Station, TX, U.S.A.) for statistical analysis.

## Additional Information

**How to cite this article**: Fedurek, P. *et al*. Sequential information in a great ape utterance. *Sci. Rep.* **6**, 38226; doi: 10.1038/srep38226 (2016).

**Publisher's note:** Springer Nature remains neutral with regard to jurisdictional claims in published maps and institutional affiliations.

## Supplementary Material

Supplementary Information
