# Supplementary material for: Sequential information in a great ape utterance
Source: Sci Rep. 2016 Dec 2;6:38226. doi: 10.1038/srep38226 (PMC5133612; doi:10.1038/srep38226)
Supplement: Supplementary Information [file srep38226-s1.pdf]

# Sequential information in a great ape utterance

Pawel Fedurek [a, c, d], Klaus Zuberbühler [a, b, c] & Christoph D. Dahl [a,e]

[a] Institute of Biology, University of Neuchâtel, Neuchâtel, Switzerland;

[b] School of Psychology and Neuroscience, University of St Andrews, Scotland (UK);

[c] Budongo Conservation Field Station, Masindi, Uganda;

[d] Max Planck Institute for Evolutionary Anthropology, Department of Primatology, Leipzig, Germany

[e] Centre for Advanced Studies, National Research University, Higher School of Economics, Moscow, Russia

Correspondence: Pawel Fedurek, Max Planck Institute for Evolutionary Anthropology, Department of Primatology, Deutscher Platz 6, D-04103 Leipzig, Germany. E-mail: fedurek@hotmail.co.uk

**<u>SUPPLEMENTARY INFORMATION</u>**

**<u>Supplementary audio data</u>**

Supplementary Audio S1: A pant hoot given by an adult male in a travelling context.

**<u>Supplementary methods</u>**

*Subjects*

From the 14 individuals, four males were excluded from analyses because they were notoriously difficult to follow (N=2 adult males), had a substantial handicap due to snare injures (N=1 adult male), or very rarely produced pant hoots (N=1 adolescent male). Table S1 shows ages and social rank of the study subjects.

Table S1: Estimated age and dominance rank (rank; 1-10) of the focal males at the onset of the study.

| Male ID | Age (years) | Rank |
|---|---|---|
| FK | 14 | 6 |
| HW | 20 | 2 |
| KT | 18 | 5 |
| KZ | 18 | 10 |
| MS | 22 | 1 |
| NK | 31 | 4 |
| PS | 15 | 9 |
| SQ | 22 | 3 |
| ZF | 31 | 8 |
| ZL | 18 | 7 |

*Social status*

Social status was established only for adult and late adolescent males, using the Elo-rating procedure. This method is based on a sequence in which interactions between individuals occur rather than on an interaction matrix. At the onset of the process each individual was given the same rating of a value 1000. After each agonistic or submissive interaction the score was updated with the winner of the interaction gaining whereas the looser losing points [1]. The number of points gained or lost by two interacting individuals was dependent on the expected outcome, which in turn depended on previous interactions between these two individuals [2]. In our study the scores were based on pant grunts (i.e. vocalisations given by males to other males that outrank them) combined with the outcomes of dyadic win-lose agonistic interactions (physical attack, chase, charge, or displacements [3,4] recorded during the study period. Since dominance relationships between male chimpanzees change frequently [5], we calculated Elo-rating scores for the following five periods separately: June to October 2013, June 2013 to May 2014, June 2013 to September 2014, June 2013 to April 2015, and May to October 2015. The Elo-rating scores were then converted into rank orders for each male (from 1 to 10, with 1 representing the highest ranking male; Table S1). The Elo-rating method has several advantages over more traditional methods such as sensitivity to short-term demography changes, effectiveness in tracking hierarchy dynamics on short-term scales, and more effective evaluation of relative hierarchy position between individuals with undecided interactions [1]. We believe that this method was especially effective in establishing dominance positions of the Sonso males, since the hierarchy was unstable throughout the study period with no clear alpha male after one of the males had lost his alpha status prior to the study period. Elo-rating scores were calculated using R v.3.1.1 (The R Foundation for Statistical Computing, Vienna, Austria).

## Supplementary references

1. Neumann, C. *et al.* Assessing dominance hierarchies: validation and advantages of progressive evaluation with Elo-rating. *Anim. Behav.* **82,** 911–921 (2011).
2. Elo, A. E. *The rating of chessplayers, past and present*. (Arco Pub., 1978).
3. Goodall, J. *The chimpanzees of Gombe: Patterns of behavior*. (Harvard University Press, 1986).
4. Muller, M. N. & Wrangham, R. W. Dominance, aggression and testosterone in wild chimpanzees: a test of the 'challenge hypothesis'. *Anim. Behav.* **67,** 113–123 (2004).
5. Gilby, I. C. & Wrangham, R. W. Association patterns among wild chimpanzees (*Pan troglodytes schweinfurthii*) reflect sex differences in cooperation. *Behav. Ecol. Sociobiol.* **62,** 1831–1842 (2008).
